# Pregnancy Gestation Impacts on HIV-1-Specific Granzyme B Response and Central Memory CD4 T Cells

**DOI:** 10.3389/fimmu.2020.00153

**Published:** 2020-02-11

**Authors:** Alexander T. H. Cocker, Nishel M. Shah, Inez Raj, Sarah Dermont, Waheed Khan, Sundhiya Mandalia, Nesrina Imami, Mark R. Johnson

**Affiliations:** ^1^Imperial College London, London, United Kingdom; ^2^Chelsea and Westminster Hospital, London, United Kingdom

**Keywords:** HIV-1, pregnancy, T-cell responses, Gag and Nef, immunity, reproduction

## Abstract

Pregnancy induces alterations in peripheral T-cell populations with both changes in subset frequencies and anti-viral responses found to alter with gestation. In HIV-1 positive women anti-HIV-1 responses are associated with transmission risk, however detailed investigation into both HIV-1-specific memory responses associated with HIV-1 control and T-cell subset changes during pregnancy have not been undertaken. In this study we aimed to define pregnancy and gestation related changes to HIV-1-specific responses and T-cell phenotype in ART treated HIV-1 positive pregnant women. Eleven non-pregnant and 24 pregnant HIV-1 positive women were recruited, peripheral blood samples taken, fresh cells isolated, and compared using ELISpot assays and flow cytometry analysis. Clinical data were collected as part of standard care, and non-parametric statistics used. Alterations in induced IFNγ, IL-2, IL-10, and granzyme B secretion by peripheral blood mononuclear cells in response to HIV-1 Gag and Nef peptide pools and changes in T-cell subsets between pregnant and non-pregnant women were assessed, with data correlated with participant clinical parameters and longitudinal analysis performed. Cross-sectional comparison identified decreased IL-10 Nef response in HIV-1 positive pregnant women compared to non-pregnant, while correlations exhibited reversed Gag and Nef cytokine and protease response associations between groups. Longitudinal analysis of pregnant participants demonstrated transient increases in Gag granzyme B response and in the central memory CD4 T-cell subset frequency during their second trimester, with a decrease in CD4 effector memory T cells from their second to third trimester. Gag and Nef HIV-1-specific responses diverge with pregnancy time-point, coinciding with relevant T-cell phenotype, and gestation associated immunological adaptations. Decreased IL-10 Nef and both increased granzyme B Gag response and central memory CD4 T cells implies that amplified antigen production is occurring, which suggests a period of compromised HIV-1 control in pregnancy.

## Introduction

Following HIV-1 infection, seropositive individuals generate specific immune responses against HIV-1 antigens that contribute to virological control (1–3). Responses to Gag and Nef have been found to dominate in early infection with increased Nef dominance being observed with decreased CD4 T-cell count, while in chronic infection the prevalence of Nef responses decrease (2–4). Elite controllers have potent enough responses to control HIV-1 viraemia, however the majority of HIV-1-infected individuals' responses are inadequate to fully suppress the virus, demonstrating disrupted CD4 to CD8 T-cell ratios and memory subset frequencies, and without antiretroviral therapy (ART) progression ensues (2, 5–8). In addition, the absolute number of Gag responding CD4 T cells decreases without ART, although the maintenance of responsive HIV-1-specific memory T-cell subsets in chronic progressors is associated with slower disease advancement, more gradual T-cell loss and control of background viral replication (9–16). In pregnancy these responses are linked to vertical transmission incidence, with higher Nef responses and interleukin-10 (IL-10) plasma concentrations associated with decreased transmission risk, though few studies have explored these relationships (17, 18).

Pregnancy studies of HIV-1 negative women have characterized gestational alterations in leukocyte populations and immune function; natural killer (NK) cell, dendritic cell (DC), granulocyte, monocyte, and T-cell subset counts change with gestation week in the peripheral blood, and increased CD4 T-cell effector memory (EM) populations have been observed (19, 20). Furthermore, peripheral anti-viral responses alter, with *in vitro* Influenza A stimulation eliciting greater activation of monocyte and DC populations from pregnant women and Influenza vaccination promoting higher interferon-γ (IFNγ) production by NK and T cells *ex vivo*, though clinical studies show pregnancy status is associated with poorer outcomes in true Influenza infection (21, 22). However, IFNγ and IL-10 responses to Cytomegalovirus, Epstein-Barr virus and other viruses are reduced in the second trimester, suggesting pregnancy immune response modulation is virus, and potentially antigen, specific (20, 23, 24).

CD4 T-cell counts in HIV-1 positive women decrease in pregnancy before restoration post-partum, and under ART pregnancy is not associated with HIV-1 disease progression (25, 26). In early HIV-1 infection IL-2 signaling is compromised which impacts the development and maintenance of T-cell memory populations, while other work suggests the degranulation capacity of CD8 cytotoxic T-cells is disrupted, and CD4 and CD8 T-cell subset frequencies are skewed even under ART (8, 27–30). Memory CD4 T-cell frequency is increased in HIV-1 positive pregnant and non-pregnant women compared to their HIV-1 negative counterparts, although detailed changes in T-cell memory subsets have not been explored between non-pregnant and pregnant HIV-1 positive women under ART (31).

Through anti-CD3 stimulation of peripheral leukocytes pregnant HIV-1 positive women have been shown to have higher IL-10 and lower TNFα and IFNγ responses than non-pregnant HIV-1 positive women (32). Physiological changes occurring during pregnancy are also known to affect ART pharmacokinetics (33). However, pregnancy's effect on HIV-1-specific responses is unknown, with work demonstrating higher acquisition of HIV-1 and other pathogens during pregnancy signifying such responses may be suppressed, suggesting control of HIV-1 and risk of transmission could be affected by gestation (34, 35). Here we sought to assess and compare Gag and Nef responses and T-cell differentiation in HIV-1 positive non-pregnant and pregnant women. Our aim was to define pregnancy's impact on the T-cell compartment and subsequent systemic HIV-1-specific functional responses that are associated with virological control of HIV-1.

## Methods

### Study Design, Ethics, Setting, and Participants

HIV-1 positive non-pregnant (PnP) and pregnant (PP) women were recruited from Chelsea and Westminster Hospital and St. Mary's Hospital, with any participant who delivered preterm excluded from analysis. Where possible blood was sampled at each trimester and delivery; two first trimester (<13 weeks gestation), 15 second trimester (13–27 weeks), 31 third trimester (>28 weeks) and 9 delivery samples were collected (median and range gestation were 8 (8), 22 (16–27), 30 (28–39), and 40 (37–40) respectively). Samples were processed within 6 h of collection, with peripheral blood mononuclear cells (PBMC) isolated in a containment level 3 laboratory. The NHS London-Chelsea Research Ethics Committee provided study approval (11/LO/0971 and 96.ND14) and clinical data collection occurred as part of standard care.

### PBMC Isolation and ELISpot Assays

Peripheral blood was collected in lithium-heparin Vacutainers (Becton Dickinson, Oxford, UK) and PBMC separated by density gradient centrifugation with Histopaque (Sigma-Aldrich, Poole, UK). Freshly isolated PBMC were resuspended to 2.5 × 10^6^ cells/ml in RPMI-1640 solution supplemented with L-glutamine, penicillin/streptomycin and human AB serum (final concentrations of 2 mM, 100 IU/ml, 100 μg/ml and 10% respectively; all Sigma-Aldrich). Polyvinylidene diflouride backed 96-well plates (Millipore, Watford, UK) were prepared with cytokine/protease specific monoclonal antibodies following manufacturer's instructions (IFNγ−3420-2A, IL-2−3445-2A, IL-10−3430-2A, granzyme B−3485-2A; Mabtech, Nacka Strand, Sweden), then 100 μl Gag, Nef (both overlapping 20 mer peptide pools, ARP788 1-22 and ARP7074 1–20 respectively; Centre for AIDS Reagents, NIBSC, UK), or PHA (Sigma) were added into separate wells (all 5 μg/ml final concentration). Non-supplemented RPMI was used as a negative control. Stimulations and controls were performed in duplicate. 2.5 × 10^5^ PBMC (100 μl) were added per well and incubated for 40 h at 37°C, 5% CO_2_ before addition of biotinylated antibodies, streptavidin and staining with AP conjugate (1706432; Bio-Rad Laboratories Ltd., Hemel Hempstead, UK). Stained plates were read on an AID automated plate reader (Oxford Biosystems Cadama, Wheatley, UK). Duplicate well means were multiplied to obtain the frequency of spot forming cells per million PBMC (SFC/1 × 10^6^ PBMC) with negative control/background subtracted from corresponding results and emerging negative values corrected to zero. Participant responses under the upper 95% confidence interval (CI) of the relevant unstimulated background were defined as non-responders.

### Flow Cytometry Methods

One to 2 × 10^6^ freshly isolated PBMC were stained with a fluorescently labeled monoclonal antibody panel to explore T-cell memory subsets. Antibodies specific to CD3, CD4, CD8, CD28, CD31, CD45RA, and CCR7 were used in tandem with a dead cell dye (Supplementary Table 1 and Supplementary Figure 1). Stem memory-like T cells (Tscm) were identified using antibodies specific to CD3, CD4, CD8, CD27, CD45RA, CD45RO, CD62-L, CD95, CD122, and CCR7, plus a dead cell dye, to stain 2–4 × 10^6^ freshly isolated PBMC (Supplementary Figure 2) (36). Isotype-matched monoclonal antibodies and fluorescence minus one (FMO) were used as controls, and a representative compensation matrix as well as gating of memory subsets based on FMO and CD62-L expression are included in Supplementary Figure 3 (37). PBMC were incubated with antibodies for 20 min at RT°C, washed with PBS, fixed in 2% paraformaldehyde solution (Becton Dickinson) and stored at 4°C until acquisition within 24 h. Samples were acquired on a BD LSR II using the FACSDiva v6.0 software (both Becton Dickinson) following both daily cytometer setup and tracking performance checks and daily cytometer setup following optimisation and longitudinal standardization steps following the Perfetto et al. protocol (38). In brief, the cytometer was calibrated to find the voltage range for stable acquisition of each detector where positive and negative peaks were well-separated, then identified a target MFI range for each detector where primary detector signal was highest with minimum spill-over into other detectors. These detector target values were used for daily cytometer setup prior to acquisition. Cytometric data analysis was undertaken on FlowJo v10.4.2 (FlowJo, Ashland, OR).

### Statistical Methods

The primary study outcome was ELISpot responses with secondary outcomes being clinical and phenotypic parameter differences between HIV-1 positive pregnant and non-pregnant women. Small group numbers (*n* < 20 participants) were anticipated and non-parametric analyses were planned as no distributional assumptions could be made. Being an exploratory study no power calculations were performed (39). Mann-Whitney *U* (two-tailed), Chi squared, and Spearman's correlation tests were used for inter-group demographic, clinical, phenotypic, and functional comparison of data collected at time of recruitment. Statistical analysis was carried out using GraphPad Prism® v7.0 (GraphPad Software Inc., California, USA), and Spearman's correlation undertaken and plotted using the psych and corrplot packages in R v3.5.2 (Supplementary Information 1) (40, 41). As correlation analysis was exploratory no correction for multiplicity was implemented to prevent increasing Type 2 error, and ELISpot data was included without implementing the 95% CI positivity threshold. Non-linear MIXED methods analysis was performed on longitudinal data of the HIV-1 positive pregnant group using SAS v9.4 (SAS Institute, Buckinghamshire, UK).

## Results

### White Blood Cell Counts Are Expanded in HIV-1 Positive Pregnancies Due to Increased Monocyte and Neutrophil Populations

Individuals were screened based on their sex, age, and HIV-1 status, with 18–45 year old HIV-1 positive women, on or commencing ART being eligible for recruitment. Women on immunomodulators or with autoimmune disorders were excluded. Eleven PnP and 24 PP women were recruited, demographic, and clinical parameters from participants time of recruitment analyzed (Table 1), with no significant difference found between group age, ethnicity, ART regimen, plasma HIV-1 RNA copies at time of recruitment, years since HIV-1 diagnosis, highest HIV-1 RNA count, days from last detectable viral load, years on ART, lymphocyte count and frequency (CD3, CD4, CD8, and CD56), nadir CD4 T-cell count, and CD4–CD8 T-cell ratio. White blood cell (WBC) count demonstrated significantly higher results in the PP women on ART, similar to those seen in pregnant HIV-1 negative women, with lymphocyte, eosinophil, and basophil absolute counts showing no change, while monocyte and neutrophil counts were increased from the PnP group (42). This partly agrees with the increased WBC and neutrophil count, and reduced lymphocyte and unchanging monocyte counts observed by Mandala et al. though the non-pregnant group in their study were newly diagnosed and 35% were 60+ years of age (43).

**Table 1:** Participant demographic and clinical parameters.

**Participant data**		**HIV-1 positive**	**HIV-1 positive**	
		**Non-Pregnant**	**Pregnant**	
		**Participants** **(PnP)**	**Participants** **(PP)**	
**Number**		**11**	**24**	
				**Chi squared (*****P-value*****)**
Ethnicity	African	10 (91%)	17 (71%)	*0.4052*
	European	1 (9%)	6 (25%)	
	Asian	0 (0%)	1 (4%)	
ART regimen	EFV	3 (27%)	6 (25%)	*0.9548*
	TFV	8 (73%)	21 (88%)	
	PI	4 (36%)	9 (38%)	
	Integrase	4 (36%)	7 (29%)	
				**Mann-Whitney** **(*P-value*)**
Age (years)		36 (28–41)	31 (25–41)	*0.0730*
Gestation (weeks)		N/A	24 (8–39)	*N/A*
HIV-1 RNA (copies/ml plasma)		<20 (<20–103)	<20 (<20–780)	*0.6232*
Years since HIV-1 diagnosis		14 (4–29)	9 (0–27)	*0.0841*
Highest HIV-1 RNA (copies/ml plasma)		33,713 (39–88,850)	30,219 (62–3,322,162)	*0.3843*
Days from last detectable HIV-1 RNA		2,395 (0–4,705)	1,204 (0–4,881)	*0.2184*
Years on ART		10 (0–17)	5 (0–26)	*0.1162*
CD3 count (cells/μl blood)		1,363 (914–1,944)	1,309 (813–2,023)	*0.9437*
CD3%		75.5 (60.3–88.7)	80.9 (67.2–88.6)	*0.2122*
CD4 count (cells/μl blood)		633 (100–962)	577 (109–1,107)	*0.8781*
CD4%		34.1 (6.6–52.5)	37.7 (9.8–50.0)	*0.8967*
CD8 count (cells/μl blood)		656.5 (390–1,066)	720 (337–1,304)	*0.7956*
CD8%		36.0 (22.7–69.9)	38.1 (26.4–70.1)	*0.2807*
CD56 count (cells/μl blood)		168(37–596)	128 (64–348)	*0.2835*
CD56%		8.8 (3.0–21.7)	8.2 (4.3–24.1)	*0.6543*
Nadir CD4 T-cell count (cells/μl blood)		301 (103–549)	275 (12–859)	*0.8624*
CD4:CD8 (CD4 count/CD8 count)		1.066 (0.094–1.779)	1.005 (0.140–1.630)	*0.7239*
x10^9^ cells/Liter blood	WBC count	5.1 (3.7–7.4)	*7*.6 (5.0–11.4)	0.0014 ^**^
	Lymphocyte count	2.0 (1.3–3.3)	1.8 (0.9–2.8)	*0.2570*
	Monocyte count	0.4 (0.2–0.5)	*0*.6 (0.4–1.1)	0.0016^**^
	Neutrophil count	2.2 (1.3–4.2)	*4*.7 (3.0–6.3)	0.0004^***^
	Eosinophil count	0.2 (0.1–0.3)	0.1 (0.1–0.7)	*0.7780*
	Basophil count	0.0 (0.0–0.1)	0.0 (0.0–0.0)	*0.3333*

*This table shows participant demographic and clinical data of non-pregnant and pregnant HIV-1 positive women and where appropriate the results of Chi squared or Mann-Whitney analysis. Median values are shown with (range) given in parentheses. Pregnant clinical data is from time of consent/first sample time-point. Italics are to differentiate p-values from data, underlined text is to highlight significantly higher result. Statistical significance was defined using p-values shown as*:

* * when p < 0.05*,

** * when p ≤ 0.01*,

*** when p ≤ 0.001, and **** when p ≤ 0.0001.

### IL-10 Response to Nef Is Reduced in HIV-1 Positive Pregnant Women

**Figure 1:**
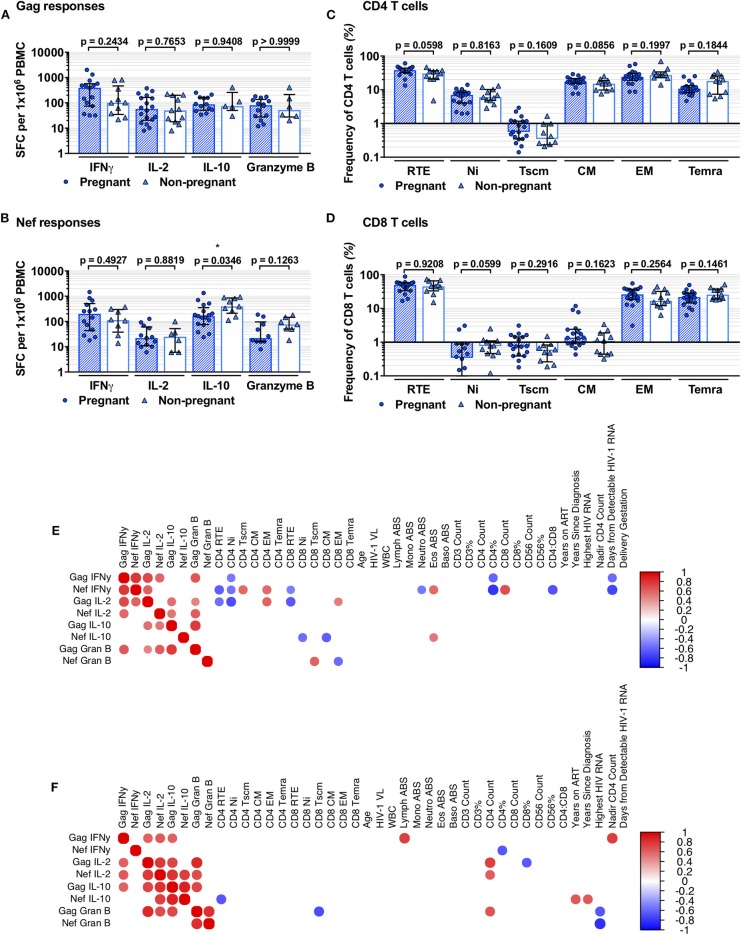
Comparison of HIV-1-specific responses and T-cell subsets of pregnant and non-pregnant HIV-1 positive women. **(A,B)** show comparison of PP and PnP group IFNγ, IL-2, IL-10, and granzyme B ELISpot spot forming cells (SFC)/1 × 10^6^ PBMC against Gag and Nef peptide pools (number per group corresponds to positive responders detailed in Table 2). **(C,D)** illustrate the frequency of RTE, Ni, Tscm, CM, EM, and Temra subset frequencies within the CD4 and CD8 T-cell compartments (for PP and PnP *n* = 18 and *n* = 11 respectively; for Tscm subset *n* = 19 and *n* = 10). Median and IQR are shown for **(A–D)**, and inter-group comparison was performed using Mann-Whitney analysis with significant differences shown as * when *p* < 0.05, ** when *p* ≤ 0.01, *** when *p* ≤ 0.001, and **** when *p* ≤ 0.0001. **(E,F)** are heatmaps of Spearman's correlation results, **(E)** showing PP group and **(F)** showing PnP group results. Circles represent significant correlations where *p* < 0.05, with positive associations in red and negative in blue. No correction for multiplicity was used.

Eleven PnP and 18 PP women had ELISpot data from time of recruitment. No difference in positive responder frequencies was observed (Table 2). Positive responder SFC/1 × 10^6^ PBMC following stimulation with Gag or Nef peptide pools, HIV-1 structural or regulatory antigens respectively, of PnP and PP participants were compared, with IFNγ, IL-2, IL-10, and granzyme B responses (pro-inflammatory, proliferative, suppressive, and cytotoxic in function) to Gag found to be similar between study groups, as were IFNγ, IL-2, and granzyme B responses to Nef (Figures 1A,B). However, the PP women demonstrated a significantly lower IL-10 response to Nef than the PnP group, suggesting pregnancy may be depressing this response.

**Table 2:** Participant ELISpot responder details.

**Participant data**		**HIV-1 positive**		**HIV-1 positive**			
		**Non-pregnant**		**Pregnant**			
		**Participants**		**Participants**			
		**(PnP)**		**(PP)**			
**Number**		**11**		**18**		**Chi squared** **(*P-value*)**	
		**Gag**	**Nef**	**Gag**	**Nef**	***Gag***	***Nef***
ELISpot responses above positivity threshold	IFNγ	10/11 (91%)	8/11 (73%)	15/18 (83%)	14/18 (78%)	*0.5659*	*0.7578*
	IL-2	11/11 (100%)	6/11 (55%)	18/18 (100%)	13/18 (72%)	*0.9999*	*0.3312*
	IL-10	5/11 (45%)	9/11 (82%)	13/18 (72%)	17/18 (94%)	*0.1494*	*0.2787*
	Granzyme B	6/11 (55%)	7/11 (64%)	14/18 (78%)	10/18 (56%)	*0.1895*	*0.6681*

This table shows participant ELISpot responses that were above threshold for positivity of non-pregnant and pregnant HIV-1 positive women, with responder frequencies compared using Chi squared analysis. Responder numbers are shown with (frequency) given in parentheses. ELISpot responder numbers are from time of consent/first sample time-point. Italics are to differentiate p-values from data. Statistical significance was defined using p-values shown as: * when p < 0.05, ** when p ≤ 0.01, *** when p ≤ 0.001, and **** when p ≤ 0.0001.

### No Significant Cross-Sectional Difference Observed Between T-Cell Memory Subsets

T-cell memory subset data acquired through flow cytometry were assessed; recent thymic emigrants (RTE; CCR7+CD45RA+CD31+), naïve (Ni; CCR7+CD45RA+CD31-), stem memory-like T cells (Tscm; CCR7+CD45RA+CD45RO-CD27+CD62-L+CD95+), central memory (CM; CCR7+CD45RA-), effector memory (EM; CCR7-CD45RA-), and terminally differentiated CD45RA expressing (Temra; CCR7-CD45RA+) T-cell subsets were identified and group frequencies compared (Supplementary Figures 1, 2 and Figures 1C,D). A non-significant increase in CD4 T-cell RTE and CM subset frequencies was observed in the PP women compared to PnP participants, indicating pregnancy may promote T-cell production and differentiation, while group frequencies of Ni, Tscm, EM, and Temra CD4 T-cell subsets were similar. We differentiated RTE from Ni T cells using CD31 expression and the frequencies we observed are similar to those of other recent studies exploring the RTE compartment (44–47). In the CD8 T-cell compartment a non-significant decrease in Ni subset frequency was observed in the PP group compared to PnP women, whereas other T-cell subsets were similar in frequency.

### IFNγ Negatively Correlated to Ni CD4 T-Cell Subset Frequency in HIV-1 Positive Pregnant Group Alone

To explore the relationships between HIV-1-specific responses and both phenotypic and clinical parameters separate Spearman's correlation analyses were performed on PnP and PP group data (Figures 1E,F). PnP women exhibited positive correlations between Gag IFNγ and Gag IL-2, Nef IL-2, Gag IL-10, lymphocyte absolute count, and nadir CD4 count (*p* = 0.0426, *p* = 0.0326, *p* = 0.0442, *p* = 0.0152, and *p* = 0.0092), while a negative association was demonstrated between Nef IFNγ and CD4% (*p* = 0.0417). The PP participants demonstrated positive IFNγ correlations between Gag IFNγ and Nef IFNγ (*p* = 0.0001), which was not seen in PnP women implying individual control of Gag and Nef IFNγ response is overridden during pregnancy, between Gag IFNγ and Gag IL-2, Nef IL-2, and Gag granzyme B responses (*p* = 0.0009, *p* = 0.0088, and *p* = 0.0031), and between Nef IFNγ and Gag IL-2, CD4 Tscm and EM subsets, eosinophil count and CD8 T-cell count (*p* = 0.0068, *p* = 0.0221, *p* = 0.0163, *p* = 0.0227, and *p* = 0.0059). Negative IFNγ relationships were observed between Gag IFNγ and CD4 Ni, CD4% and days from detectable HIV-1 RNA (*p* = 0.0392, *p* = 0.0260, and *p* = 0.0123), showing differentiated HIV-1 antigen associations from the PnP women. Further negative correlations were observed between Nef IFNγ and CD4 RTE and Ni subsets, CD8 RTE subset, neutrophil count, CD4%, CD4–CD8 ratio, and days from detectable HIV-1 RNA (*p* = 0.0089, *p* = 0.0303, *p* = 0.0497, *p* = 0.0426, *p* = 0.0005, *p* = 0.0085, and *p* = 0.0005), suggesting this response may have a disproportionate impact on the T-cell compartment in pregnant women.

### Positive Association Between IL-2 Response and CD4 T-Cell Count Missing in HIV-1 Positive Pregnant Women

The PnP group exhibited positive relationships between Gag IL-2 and Nef IL-2, Gag IL-10, Gag granzyme B, and CD4 T-cell count (*p* = 0.0041, *p* = 0.0101, *p* = 0.0010, and *p* = 0.0111), and between Nef IL-2 and Gag IL-10, Nef IL-10, Gag granzyme B and CD4 T-cell count (*p* = 0.0022, *p* = 0.0073, *p* = 0.0173, and *p* = 0.0499), while a negative correlation was found between Gag IL-2 and CD8% (*p* = 0.0425). Positive PP group IL-2 correlations were found between Gag IL-2 and Gag IL-10, Gag granzyme B, and both CD4 and CD8 EM subsets (*p* = 0.0172, *p* = 0.0448, *p* = 0.0139, and *p* = 0.0486), potentially suggesting an increased Gag-responsive T-cell memory population compared to the PnP women, and between Nef IL-2 and both Gag IL-10 and granzyme B responses (*p* = 0.0226 and *p* = 0.0051). Negative IL-2 relationships were seen between Gag IL-2 and both CD4 RTE and Ni subsets, as well as CD8 RTE subset (*p* = 0.0263, *p* = 0.0025, and *p* = 0.0058), showing further discordance in both the antigen response T-cell restoration.

### Negative Relationships Between Nef IL-10 Response and Both Ni and CM CD8 T-Cell Subsets Observed in HIV-1 Positive Pregnant Cohort

Positive associations for PnP participants were found between Gag IL-10 and both Nef IL-10 and Gag granzyme B responses (*p* = 0.0003 and *p* = 0.0111), and between Nef IL-10 and both years on ART and years since HIV-1 diagnosis (*p* = 0.0278 and *p* = 0.0326), whereas a negative relationship was observed between Nef IL-10 and CD4 RTE subset frequency (*p* = 0.0260). For the PP group positive correlations were observed between Gag IL-10 and Gag granzyme B responses (*p* = 0.0001) and between Nef IL-10 and eosinophil count (*p* = 0.0468), while negative relationships were found between Nef IL-10 and CD8 Ni and CM T cells (*p* = 0.0227 and *p* = 0.0099), indicating the impact of IL-10 action on CD4 and CD8 T-cell subsets may alter during pregnancy.

### Non-pregnant HIV-1 Positive Women Alone Exhibited an Inverse Correlation Between Granzyme B Responses and Highest Recorded HIV-1 RNA

The PnP group showed positive correlations between Gag granzyme B response and both Nef granzyme B and CD4 T-cell count (*p* = 0.0019 and *p* = 0.0367), with negative associations found between Gag granzyme B and both CD8 Tscm subset and highest HIV-1 RNA (*p* = 0.0180 and *p* = 0.0345), and between Nef granzyme B and highest HIV-1 RNA (*p* = 0.0031). In the PP women Nef granzyme B response correlated positively with CD8 Tscm subset and negatively with CD8 EM population (*p* = 0.0153 and *p* = 0.0188), suggesting the earlier CD8 memory T cells generated by antigenic stimulation are more reactive during pregnancy. While two of the PnP group had detectable plasma HIV-1 RNA (Supplementary Figure 4) no correlation was observed between HIV-1 viral load and any functional response.

### Longitudinal Responses to Gag and Nef

**Figure 2:**
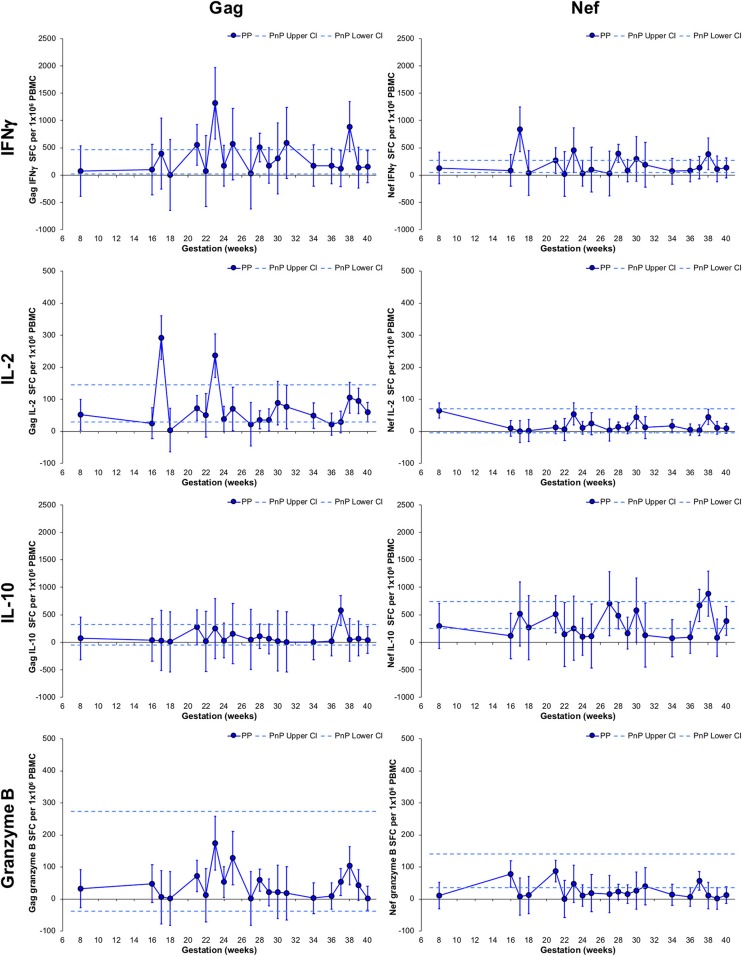
Longitudinal assessment of HIV-1-specific responses of pregnant HIV-1 positive women. The left-hand side graphs show longitudinal changes in Gag response and the right-hand side graphs illustrate changes in Nef response in HIV-1 positive pregnant women (*n* = 49, median 3 per participant). From top to bottom IFNγ, IL-2, IL-10, and granzyme B ELISpot responses are shown. The straight dashed lines show the upper and lower 95% CI for the HIV-1 positive non-pregnant group, which were assessed at a single time-point (*n* = 11). Longitudinal assessment of the HIV-1 positive pregnant participants was undertaken using non-linear MIXED methods analysis, with point estimates and 95% CI bars shown.

Non-linear MIXED methods analysis was performed on PP group longitudinal follow-up samples (*n* = 57, median 3 per participant) to describe parameter development with gestation week. Point estimates significantly higher or lower than their neighbors are defined as peaks or troughs. Those that significantly differ from non-neighboring point estimates describe a gestational increase or decrease (Figure 2). PnP cross-sectional 95% CI ranges were included for comparison.

Longitudinal PP women's Gag and Nef IFNγ responses did not deviate from the CI range of the PnP group and demonstrated a peak at 23 and 17 weeks, respectively. Neither Gag nor Nef IL-2 responses significantly changed compared to the PnP participants, while IL-2 Gag responses exhibited peaks at 17 and 23 weeks. No variation in Gag or Nef IL-10 responses from the PnP group range was observed in the PP cohort, and a peak at 37 weeks was observed in IL-10 Gag responses. While remaining within the PnP CI range, a significant increase from 17 to 23 weeks and decrease from 23 to 40 weeks was demonstrated by PP participants' Gag granzyme B responses, with PP Nef granzyme B responses exhibiting a decrease from 16 to 40 weeks and a lower response than PnP women at 39 weeks, implying gestation influences granzyme B secretion against both antigens. Participants with detectable viral plasma RNA during the Gag granzyme B 23 week peak all demonstrated decreasing viral loads from earlier time-points suggesting this transiently increased response was not directly linked to peripheral HIV-1 RNA levels (Supplementary Figure 4). Correlation analysis of viral load against either Gag or Nef granzyme B responses from a combination of pregnant and non-pregnant participants who demonstrated detectable plasma HIV-1 RNA during the study found no association between these parameters (Supplementary Figure 5).

### Longitudinal Changes to the CD4 T-Cell Compartment

**Figure 3:**
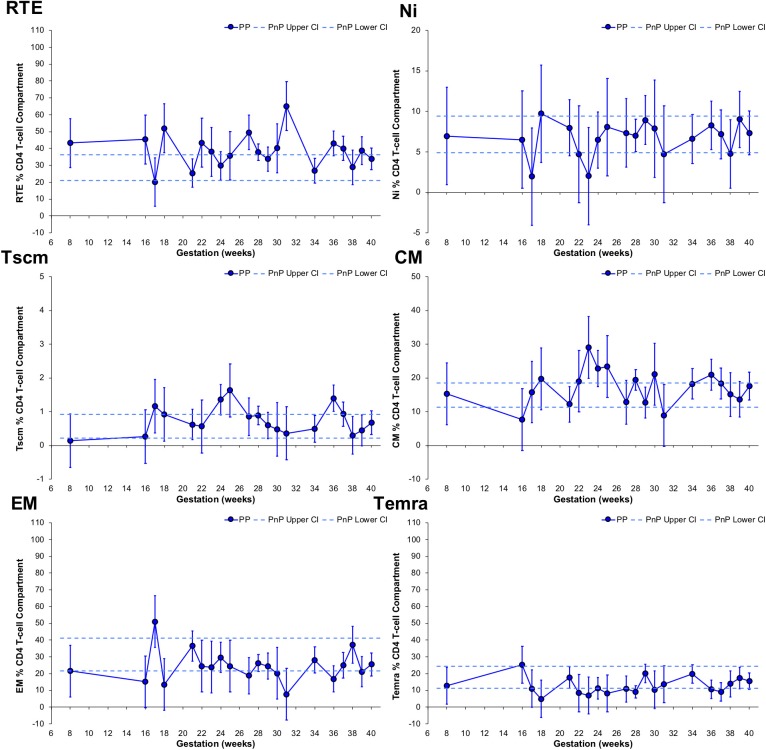
Longitudinal analysis of subset frequencies within the CD4 T-cell compartment of pregnant HIV-1 positive women. The above graphs show the longitudinal change in RTE, Ni, Tscm, CM, EM, and Temra CD4 T-cell frequencies in HIV-1 positive pregnant women (*n* = 52, median 3 samples per participant; for Tscm *n* = 51, median 3 samples per participant). The straight dashed lines show the upper and lower 95% CI for the HIV-1 positive non-pregnant group, which were assessed at a single time-point (*n* = 11; for Tscm *n* = 10). Longitudinal assessment of the HIV-1 positive pregnant participants was undertaken using non-linear MIXED methods analysis, with point estimates and 95% CI bars shown.

Longitudinal changes in CD4 T-cell subsets were also explored (Figure 3). The RTE T-cell subset frequency in PP women was significantly higher than PnP participants at 18, 27, and 31 weeks gestation, and demonstrated a trough at 34 weeks. No change from PnP women was seen in either Ni or Tscm subsets, while a peak was seen at 36 weeks in the PP Tscm population. The CM subset frequency was higher in PP women than PnP participants at 23 weeks and a significant increase from 16 to 23 weeks and decrease from 23 to 39 weeks was observed, suggesting increased proliferation of this subset peaking in the middle of pregnancy. EM and Temra subset frequencies did not alter from the PnP group range, while in PP women a peak at 17 weeks and a significant decrease from 21 to 36 weeks was found in the EM T-cell population, potentially indicating gestational restriction of differentiation is occurring.

### Longitudinal Changes to the CD8 T-Cell Compartment

**Figure 4:**
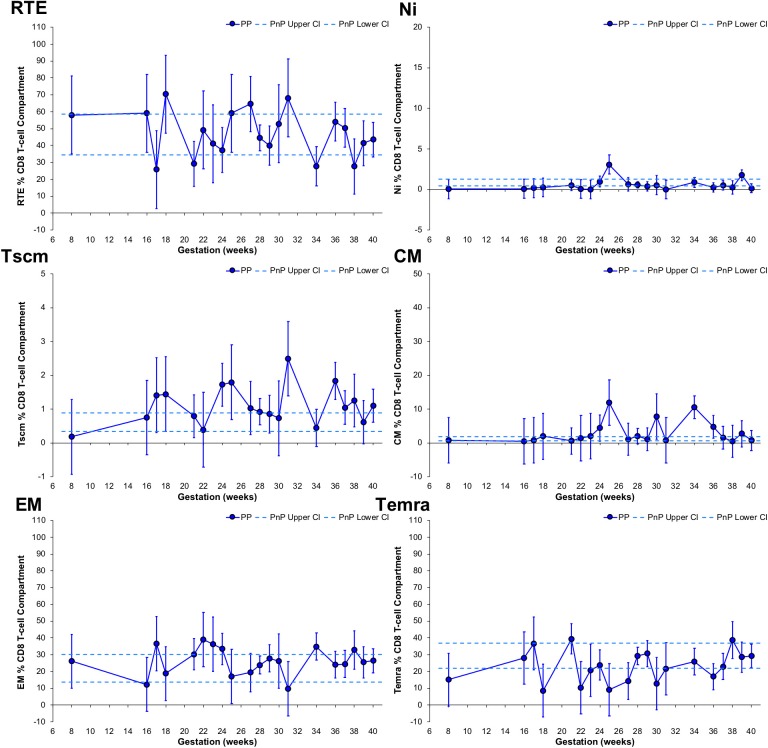
Longitudinal analysis of subset frequencies within the CD8 T-cell compartment of pregnant HIV-1 positive women. The above graphs show the longitudinal change in RTE, Ni, Tscm, CM, EM, and Temra CD8 T-cell frequencies in HIV-1 positive pregnant women (*n* = 52, median 3 samples per participant; for Tscm *n* = 51, median 3 samples per participant). The straight dashed lines show the upper and lower 95% CI for the HIV-1 positive non-pregnant group, which were assessed at a single time-point (*n* = 11; for Tscm *n* = 10). Longitudinal assessment of the HIV-1 positive pregnant participants was undertaken using non-linear MIXED methods analysis, with point estimates and 95% CI bars shown.

CD8 T-cell subset frequency changes with gestation were assessed, with RTE T-cell frequency of PP participants unchanged from the PnP group range, demonstrating a peak at 18 weeks, a trough at 34 weeks, and a significant increase from 21 to 31 weeks gestation (Figure 4). Peaks at 25 and 39 weeks were found in PP participants' Ni T-cell subset, the former being above the PnP cohort range, while the PP group's Tscm population showed higher frequencies than PnP women at 24, 31, and 36 weeks gestation with a trough observed at 34 weeks. The CM T-cell subset in PP women at 25 and 34 weeks were higher than the PnP range, and a significant increase from 21 to 25 weeks, decrease from 25 to 28 weeks, increase from 28 to 34 weeks, and decrease from 34 to 37 weeks were observed, indicating gestation also impacts the differentiation of the CD8 compartment. PnP and PP group ranges overlapped for both EM and Temra subsets, and in PP women a trough at 31 weeks was found in the EM population, and in the Temra subset a peak at 21 weeks and increase from 36 to 38 weeks gestation was demonstrated.

## Discussion

With this work we aimed to define pregnancy and gestation related changes to HIV-1 responses, and associated alterations in T-cell subsets to determine if pregnancy impacts on the adaptive control of HIV-1 infection. Here we address the question of how pregnancy affects the control of HIV-1 infection and sought to answer this in the context of ART treated women. Our study demonstrates for the first time that pregnancy decreases IL-10 response to Nef, transiently increases Gag granzyme B response in tandem with CD4 T-cell CM frequency, as well as decreasing CD4 EM T-cell population and altering CD8 CM T-cell frequencies of HIV-1 positive women.

Recent studies have highlighted the importance of the functional control of HIV-1 and the relevance of memory T-cell maintenance and reconstitution in disease progression (3, 30). Disrupted HIV-1-specific responses may impact HIV-1 control and potentially instigate periods of immune activation that in HIV-1 negative pregnancies are associated with preterm birth and fetal growth restriction (48). Through comparison of peripheral blood HIV-1-specific immune responses between ART treated pregnant and non-pregnant groups, we demonstrated that the suppressive IL-10 response against Nef is significantly reduced in the pregnant cohort. The magnitude of these IL-10 responses and the difference between the groups were similar to the difference in IFNγ Gag responses found between HIV-1 controllers and chronic progressors (49). No alteration in IFNγ or IL-2 secretion was observed, indicating ability to initiate pro-inflammatory and proliferative responses against HIV-1 are maintained in PP women on ART. However, IL-10 response to Nef was decreased and direct correlations of Gag and Nef IFNγ, IL-2, IL-10, and granzyme B responses were reversed with pregnancy status, suggesting the maintenance of Gag and Nef-specific T-cell populations is affected by pregnancy. This could be caused by changes in HIV-1-specific T-cell subset stimulation related to changes in the proportion of antigen production suggesting a potential switch from latent to activated HIV-1 reservoir cells (50, 51). This antigenic load shift is reminiscent of the changes observed in HIV-1 positive individuals during treatment interruption (52).

Our exploration of gestational HIV-1 response changes using non-linear MIXED methods analysis, uniquely suited to describing longitudinal developments, which has not been done previously implies that gestation alters HIV-1 control and suggests low level viraemia may be driving the adaptations we have observed. The transient increase in granzyme B response hints that priming of CD8 T cells by Gag presentation is occurring. This would mean more Gag is present, therefore HIV-1 control is suboptimal during pregnancy allowing increased HIV-1 peptide generation. In untreated PP women of African ethnicity a non-significant increase in HIV-1 RNA was observed with gestation, and numerous pharmacokinetic studies indicate pregnancy negatively affects ART levels (53–56). In an ART interruption study increases in both Gag and Nef CD8 T-cell responses were found with rebounding viraemia (52). Together these findings suggest a low-level increase in HIV-1 activity has caused these functional changes.

Following this we explored the phenotype of peripheral CD4 and CD8 T-cell populations, focussing on their differentiation state to investigate if pregnancy preferentially drives the proliferation of antigen naïve or experienced subsets, which highlighted a potential increase in CD4 CM T-cell frequency in the pregnant group compared to the non-pregnant. The temporary gestational increase in CM and decrease in EM T-cell subsets indicates a change in immunological homeostasis in HIV-1 positive pregnancy. Transiently increased endogenous STAT5 phosphorylation in Ni T cells, and separately decreased IL-2 plasma concentrations from before to after 25 weeks gestation, have been found in HIV-1 negative pregnancies, suggesting IL-2 signaling plays a role in gestation associated changes (57, 58). Low-level Gag presentation may be co-opting the IL-2 pregnancy stimulation of the Ni T-cell subset by changing the immunological environment, driving the differentiation of CM cells (59). Lower plasma IL-2, IL-6 and TNFα have been observed in PP women compared to an HIV-1 negative pregnant group, and the gestational decrease in CD4 EM subset frequency suggests stimulation is not driving T-cell differentiation through peripheral inflammation, implying the potential promotion, and/or priming of HIV-1-specific cells is occurring in a immunologically privileged site (60).

Our findings raise two primary concerns; whether during the period of increased Gag-specific granzyme B response ART treated PP women are at increased risk of disrupting immunological balance supporting pregnancy if control of HIV-1 is reduced, and if there is potential disruption on HIV-1 control at immune privileged sites which may not be identified through peripheral blood viral load testing. While the first may affect pregnancy health, the latter could impact on transmission risk. All participants were on ART, which may mask potential pregnancy related fluctuations in the functional control of HIV-1, and it is possible the small number of women who became undetectable during the study may have influenced the longitudinal changes we found. ART commencement and decreasing viral RNA are associated with reduced HIV-1-specific functional responses, suggesting the transient increase observed in granzyme B response to Gag was not due to these detectable women (61). The incongruent timing of their longitudinal plasma HIV-1 RNA fluctuations and the lack of association between HIV-1 viral load and ELISpot responses further support this. However, being a preliminary study we cannot rule out a potential influence of plasma HIV-1 RNA on HIV-1-specific functional changes; it is possible ART-induced decreasing viral loads may promote recovery of Gag-specific CD8 T cells, improving their granzyme B response, while Nef-specific CD8 responses may decrease with viral load. Positive associations observed between PD-1 expressing HIV-1-specific CD8 T cells and HIV-1 viral load could suggest decreased viral load after ART initiation reduces CD8 T-cell exhaustion, in turn benefitting granzyme B responses which have been shown to be inversely correlated to viral load and cellular pro-viral DNA (62, 63). Either way, our results highlight an important aspect of HIV-1 positive pregnancy that deserves further exploration in larger cohorts.

While we have explored responses through ELISpot assays, we were limited in the volume of blood we could take longitudinally from pregnant women and so were unable to assess if the changes observed correspond with proliferation of antigen-specific T-cell subsets, although the relevance of these responses alone have been demonstrated through the comparison of HIV-1 controllers to chronic progressors, and their correlation to both T-cell count and HIV-1 RNA (15, 16, 49). We have also assumed that the observed changes in HIV-1 responses are antigen specific and driven by alteration in the production and presentation of antigen, although it is possible this could be caused by pregnancy induced shifts in Th1, Th2, and Treg T-cell proportions which were not measured. Future comparison of paired pre-pregnancy to pregnancy samples, comparison of other anti-viral responses in HIV-1 negative pregnant women as well as assessment of helper T-cell subset changes may better define the influence of pregnancy on immune function in PP women on ART. Our study followed an exploratory design so follow up work is required to confirm these findings. Nevertheless, our results provide evidence that pregnancy associated immunological developments differentially affect systemic HIV-1-specific T-cell function that may impact on viral control, which is especially relevant as these responses have been linked to vertical transmission risk.

## Data Availability Statement

The datasets generated for this study are available on request to the corresponding author.

## Ethics Statement

The studies involving human participants were reviewed and approved by NHS London-Chelsea Research Ethics Committee. The patients/participants provided their written informed consent to participate in this study.

## Author Contributions

AC performed the experiments. IR, SD, and WK enabled recruitment and sample acquisition. AC and SM analyzed the data. AC, NS, NI, and MJ have both written the article and designed and executed the study.

### Conflict of Interest

The authors declare that the research was conducted in the absence of any commercial or financial relationships that could be construed as a potential conflict of interest.
