# Supplementary material for: Pregnancy Gestation Impacts on HIV-1-Specific Granzyme B Response and Central Memory CD4 T Cells
Source: Front Immunol. 2020 Feb 11;11:153. doi: 10.3389/fimmu.2020.00153 (PMC7027986; doi:10.3389/fimmu.2020.00153)
Supplement: Supplementary file 1 [file Presentation_1.pdf]

Abstract Breakdown for Non-Specialists:

"Pregnancy gestation impacts on HIV-1-specific granzyme B response and central memory CD4 T cells"

An accompaniment to the published paper Abstract for those who do not specialise in HIV and Immunology

Also known as:
"The immune response that can kill cells that are infected with HIV, as well as some of the memory immune cells that can both be infected by and respond to HIV, change with the gestation week of pregnancy"

Research themes:

- The interaction between pregnancy and the maternal immune system
- The immune responses and memory of the human immunodeficiency virus (HIV, or HIV-1)

What is the point:
The immune response of pregnant women has been found to change throughout their pregnancy. This can affect how well they can respond to potential pathogenic challenges (infections by bacteria or viruses for example). If the immune response is insufficient it may not be able to prevent a pathogenic infection which could negatively affect the health of the pregnant women or fetus. If the immune response is too extreme it may disrupt the immune balance that is necessary to a successful pregnancy and could cause pregnancy complications such as early labour. These changes seem to be different for different pathogens and how responses against HIV change is not yet fully understood. This study aimed to understand how pregnancy affected the responses against HIV throughout the duration of pregnancy.

Objective of research:

- To understand how being pregnant can affect the responses that can help control HIV
- To understand how HIV responses are affected at different times during pregnancy
- To understand how the immune cells relevant to HIV responses and infection change with pregnancy

Key concepts:

Human immunodeficiency virus (HIV, or HIV-1) – HIV is a retrovirus meaning it infects cells permanently by integrating its DNA into that of the infected cell. This means the infected cell will produce HIV and no longer function as normal. The mechanism that HIV uses to enter cells relies on a molecule called CD4 which is present on the outside cell surface of some T cells.

T-cell – T cells are an immune cell type that are important in immunological memory. When they first recognise a pathogen (an infectious bacteria or virus) they respond by creating a large population of T cells that recognise specific molecules that are associated with the pathogen which then survive long-term and form a population of memory cells that can recognise and react against the pathogen in the future. T cells are split into two populations based on their outside cell surface molecules; those that have CD4 present are CD4 T cells that are important in controlling other immune cells and their responses. T cells with CD8 present are CD8 T cells that are very efficient at recognising cells infected with viruses and killing them.

T-cell memory – The function and outside cell surface molecules of T cells are affected by how recently T cells have interacted with the specific molecules that is associated with the pathogen (an antigen) they respond to. T cells are released into the blood from the Thymus (known as recent thymic emigrants at this point) and develop into naïve T cells. When they first interact with the antigen they recognise they change from naïve cells to activated cells and form memory subsets. A small subset called stem-cell memory like T cells continuously generates memory T cells that recognise the same antigen and maintains T-cell memory. Central memory T cells are a larger population of memory cells that when activated generate effector memory T cells that are more effective at responding to pathogens. When effector cells are activated for long enough they mature into terminally differentiated T cells.

Immune responses – There are a huge number of ways immune cells can respond to pathogens. Here we focussed on ones that are linked to inflammation (activating immune cells), proliferation (causing immune cells to replicate into larger numbers), suppression (controlling the immune response so it does not become damaging), and cytotoxic (causing direct killing of infected cells). Interferon-gamma (IFNγ) is a molecule that causes inflammation, interleukin-2 (IL-2) is a molecule that causes proliferation, interleukin-10 (IL-10) is a molecule that causes suppression, and granzyme B is a molecule that causes cytotoxicity.

Gag and Nef – These are HIV molecules that can be recognised by T cells and so are important antigens in HIV responses. Gag is a molecule that is important in the structure of the HIV virus, whereas Nef is a molecule that is important in controlling the internal machinery of the HIV infected cell. Cells can signal that they are infected with HIV by presenting these antigens for other immune cells to recognise and respond to. When HIV is controlled by anti-retroviral drugs these prevent HIV from being created by infected cells and so very few of these antigens are produced to be presented by infected cells.

Transmission – Transmission refers to the spreading of a pathogen, in this case HIV. Horizontal transmission would be from person-to-person, while vertical transmission means mother-to-child transmission of infection.

Anti-retroviral therapy (ART) – ART drugs control HIV by preventing the virus from creating more copies of itself and from infecting new cells. These drugs achieve this by blocking the virus lifecycle, preventing the virus from making the cell it has infected produce virus molecules, such as Gag and Nef, that are needed to produce more virus.

ELISpot – An ELISpot is an experimental technique that allows the counting of the number of immune cells that are responding to a stimulant. In this study the stimulants used were HIV antigens Gag and Nef. The responses that were counted were IFNγ, IL-2, IL-10 and granzyme B. We used freshly isolated immune cells as these responses should be similar to the cells that are circulating in the blood. Some studies are unable to look at fresh immune cell responses and instead freeze immune cells to use in experiments at a later date. These frozen cells can be revived and used for experiments like ELISpot assays but the freezing and thawing of the cells may change their responses and may be less representative of the responses of cells in the blood.

Flow cytometry – This is a technique used to identify the molecules present on immune cells (known as their phenotype). Knowing the molecules present on the immune cell surface allows the identification of the immune cell type, such as identifying if it is a CD4 or a CD8 T-cell, and can be used to tell what memory subset a cell fits into. This technique is used to look at thousands of cells from each blood sample taken and from the results it is possible to work out the percentage/frequency of cell populations and compare them between study groups.

Non-parametric statistics – If we expected the data we got from our experiments to be equally varied we would use parametric statistics to compare the groups as this would assume that the group data would vary in the same way and was normally distributed. In studies with large participants numbers (ie. hundreds per group) it can often be assumed that the data will vary in a predictable way as so many participants are included, meaning abnormal outliers do not affect the results. However, in smaller studies as there are less participants to look at the data is less predictable and so we cannot assume it will be distributed normally. It is harder to tell if data points are normal or if they are potentially abnormal outliers. This means we used non-parametric statistics as these methods allow group comparisons without assuming the group data will be equally varied. Where we compared a single point of data from each group participant this was a cross-sectional comparison; where we looked at multiple samples from the pregnant group participants this was a longitudinal comparison.

Gestation – This refers to the time point in pregnancy. Pregnancy lasts for around 40 weeks gestation; the first trimester is from 0 to 12 weeks gestation, the second trimester from 13 to 27 weeks gestation, and the third trimester from 28 weeks until the end of pregnancy.

Peripheral blood mononuclear cells – This is a more descriptive way to say immune cells present in blood.

Correlation of HIV responses to clinical parameters – We correlated the results we found from our ELISpot experiments where we looked at HIV responses to the matched clinical data for each participant. This is a statistics method that can identify if two things change together. If two parameters are found to change together, or if one parameter always goes up when the other goes down, this can suggest they may be linked in some way. This does not however prove that one parameter changing causes the other to change. Clinical parameters refers to data that is collected as part of the participants normal/routine care and is important for their clinical care team to monitor to understand their health in relation to HIV and pregnancy.

Study results:

Cross-sectional comparison identified decreased IL-10 Nef response in HIV-1 positive pregnant women compared to non-pregnant. This indicated that the suppressive IL-10 response against the HIV antigen Nef is reduced in pregnancy.

Correlations exhibited reversed Gag and Nef cytokine and protease response associations between groups. IFNγ, IL-2 and IL-10 are collectively called cytokines, and granzyme B is a protease. Different levels of these immune responses can be found against different antigens, meaning for example that IL-10 Gag responses may not increase when IL-10 Nef responses increase. This result indicates that the relationship between these responses has been altered by pregnancy, and as the change in relationship is occurring in all responses it suggests the common denominator, the antigens, have been affected by pregnancy.

Longitudinal analysis of pregnant participants demonstrated transient increases in Gag granzyme B response and in the central memory CD4 T-cell subset frequency during their 2nd trimester, with a decrease in CD4 effector memory T cells from their 2nd to 3rd trimester. The temporary increase in granzyme B response to Gag suggests that during this increase there is more Gag antigen to activate the T cells that recognise Gag and respond by secreting granzyme B. The temporary increase in central memory cells indicates the T-cell population is being activated during this same period, while the overall decrease in effector memory suggests it is only the earlier memory population that is being stimulated.

Study conclusions:

Gag and Nef HIV-1-specific responses change with pregnancy time-point, coinciding with relevant T-cell phenotype and gestation associated immunological adaptations. Decreased IL-10 Nef, and both increased granzyme B Gag response and central memory CD4 T cells implies amplified antigen production which suggests a period of compromised HIV-1 control in pregnancy.

Our results provide evidence that pregnancy associated immunological developments differentially affect systemic HIV-1-specific T-cell function that may impact on viral control, which is especially relevant as these responses have been linked to vertical transmission risk.

How this work fits in with other research:

Our findings raise two possible issues: the period of increased Gag-specific granzyme B response HIV positive pregnant women could suggests the control of HIV may be reduced as the immune response against the HIV Gag molecule is increasing, ie. more HIV is present to create/boost this response. If HIV is not as well controlled during pregnancy this could instigate immune responses against HIV that may negatively affect the immune support of pregnancy. Recent studies looking into the level of HIV ART drugs in pregnant women have found that the physiological changes associated with pregnancy can reduce drug levels, though have not found this necessarily affects HIV control. Looked at together with our work this may mean the reduced HIV anti-viral drug levels are controlling HIV a little less than when not pregnant which could be causing the increased response against HIV that we found. Importantly, this may mean the time when the HIV response is highest may be a period when there is more risk of HIV responses being disruptive to pregnancy.

Important limitations:

All the participants in our study were on HIV anti-retroviral drugs which controls HIV levels and so the changes we saw in HIV positive pregnant HIV responses are not necessarily the same as those of HIV positive pregnant women who are not on HIV anti-retroviral drugs.

The pregnant and non-pregnant women we were comparing were similar but we cannot be completely certain that if the non-pregnant women were pregnant we would see the same changes in HIV response and T-cell frequency. If we had compared the HIV responses of the same women when they were not pregnant to their responses once they were pregnant our findings would be more conclusive.

Acknowledgements:

Thank you to Pamela Cocker and James McParland who read, corrected and much improved this abstract breakdown.
