# Supplementary material for: Pregnancy Gestation Impacts on HIV-1-Specific Granzyme B Response and Central Memory CD4 T Cells
Source: Front Immunol. 2020 Feb 11;11:153. doi: 10.3389/fimmu.2020.00153 (PMC7027986; doi:10.3389/fimmu.2020.00153)
Supplement: Supplementary file 2 [file Data_Sheet_1.pdf]

Supplementary section

Supplementary Table 1 – Fluorescently labelled monoclonal antibodies This table details the fluorescently labelled monoclonal antibodies clone, manufacturer, product code and titrated volume used in this study. Antibodies used were from either Becton Dickinson (BD), BioLegend (BL), or Thermo Fisher (TF).

Fluorochrome	Marker	Clone	Supplier and Product Code	Research Resource Identifier	Volume per $1x10^6$ PBMC/100µl
BV650	CD45RA	HI100	BD; 563963	RRID:AB_2738514	3µl
BV605	CD4	RPA-T4	BD; 562658	RRID:AB_2744420	3µl
BV421	CD28	CD28.2	BD; 562613	RRID:AB_2737676	5µl
BV421	CD122	TU27	BL; 339010	RRID:AB_2561835	2µl
Amcyan	Aqua Live/Dead dye	-	TF; L34957	-	1µl
FITC	CD27	M-T271	BL; 356404	RRID:AB_2561788	2µl
PE	CD31	WM56	BD; 555446	RRID:AB_395839	20µl
PE	CD62-L	DREG-56	BL; 304806	RRID:AB_314466	3µl
APC	CD4	RPA-T4	BD; 555349	RRID:AB_398593	20µl
APC	CD95	DX2	BD; 558814	RRID:AB_398659	10µl
PE-CF594	CD45RO	UCHL1	BD; 562299	RRID:AB_11154398	3µl
PE-Cy7	CCR7	3D12	BD; 557648	RRID:AB_396765	3µl
PerCP-Cy5.5	CD3	SK7/Leu-4	BD; 332771	-	3µl
APC-H7	CD8	SK1	BD; 560179	RRID:AB_1645481	3µl

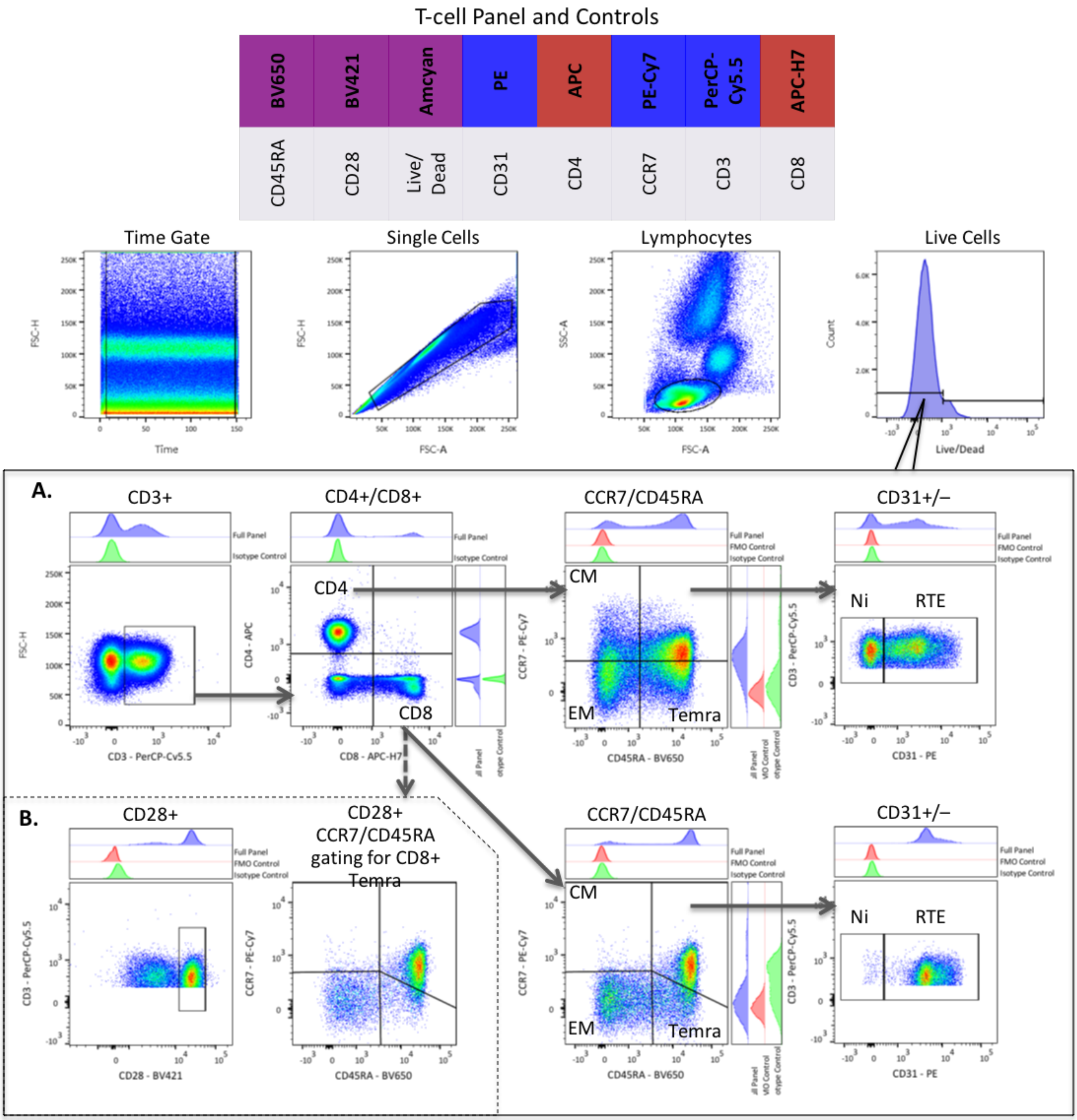

Supplementary Figure 1 – Gating strategy for T-cell subset identification This figure identifies T-cell subsets through gating flow cytometry data by time, single cells and size as measured by FSC and SSC values followed by exclusion of dead cells. **A.** Shows the identification of T cells through CD3+ expression, then helper (CD4+) and cytotoxic (CD8+) subsets. Memory/maturation stages of CD4 and CD8 T-cell populations are then defined; recent thymic emigrants (RTE; CCR7+CD45RA+CD31+), naïve (Ni; CCR7+CD45RA+CD31–), central memory (CM; CCR7+CD45RA–), effector memory (EM; CCR7–CD45RA–), and terminally differentiated CD45RA expressing T cells (Temra; CCR7–CD45RA+). **B.** Illustrates gating validation for CD8+ T cells; the Temra population gate (CCR7–CD45RA+) was defined by gating on CD28+ CD8 T cells only as expression of this surface marker is lost in the maturation to this stage in cytotoxic T cells. Control comparisons are shown in accompanying axis histogram overlays with the full panel on top in blue, FMO controls in the middle in red, and isotype controls on the bottom in green.

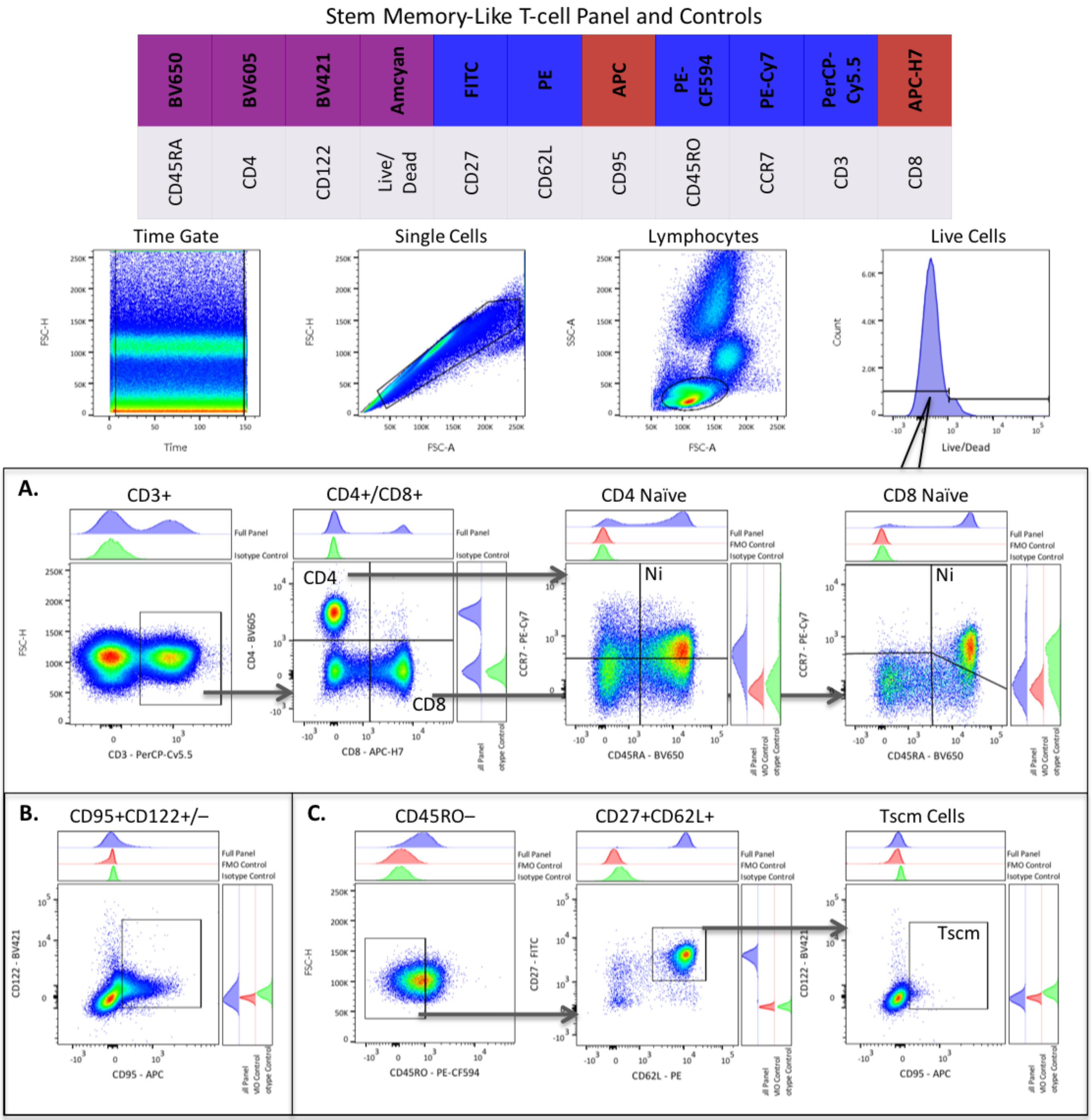

Supplementary Figure 2 – Gating strategy for Tscm cell identification Stem memory-like T-cell (Tscm) subsets were identified through gating flow cytometry data by time, single cells and size as measured by FSC and SSC values followed by exclusion of dead cells. **A.** Shows the identification of T cells through CD3+ expression, then helper (CD4+) and cytotoxic (CD8+) subsets, and finally defines naïve T cells (Ni) as CCR7+CD45RA+. **B.** Shows the CD95+CD122+/– gate created on total T cells to validate later Tscm gating. **C.** Shows gating for either CD4 or CD8 Ni subsets, where the Tscm population is defined as CD45RO–CD27+CD62L+CD95+. Control comparisons are shown in accompanying axis histogram overlays with the full panel on top in blue, FMO controls in the middle in red, and isotype controls on the bottom in green.

Tscm Compensation Matrix

Show All	APC-A	APC-H7-A	AmCyan-A	BV 421-A	BV 605-A	BV 650-A	FITC-A	PE-A	PE-CF594-A	PE-Cy7-A	PerCP-Cy5-5-A
APC-A	100	22.8905	-0.1669	-0.1441	0.1078	23.2737	0	0	0.0359	0.4133	1.8011
APC-H7-A	0.5478	100	-0.1884	0.8208	0.0456	0.1597	0	0	0	1.8963	0.0228
AmCyan-A	0.6646	0.4652	100	16.4611	9.9727	3.4661	4.9727	3.4704	2.7978	0.2658	1.0636
BV 421-A	0	0	6.7578	100	0.1175	0.0238	0.0012	0	0	0	0
BV 605-A	0.0605	0.0181	0.8112	10.3209	100	43.4319	0	0.6125	7.7585	0.3994	1.6465
BV 650-A	5.1059	1.5664	1.9995	21.7998	14.6404	100	0.0241	0.0386	0.2268	0.0964	0.6197
FITC-A	0	0	6.4321	0	0.2673	0.0562	100	22.1222	7.9507	0.1265	0.7043
PE-A	0.0046	0.0046	0.1541	0	3.4104	0.795	0.7673	100	41.9384	0.7398	4.8722
PE-CF594-A	0.098	0.0413	0	0	7.6695	2.3584	0.1045	5.6713	100	3.8149	19.9838
PE-Cy7-A	0	8.2803	0	0	0.0533	0.0097	0.2328	1.7266	0.7566	100	0.2473
PerCP-Cy5-5-A	1.8322	5.7267	-0.3437	1.0265	0.0555	5.9014	0	0.0555	0.2218	26.4071	100

CD4 and CD8 Dot Plot FMO Gating

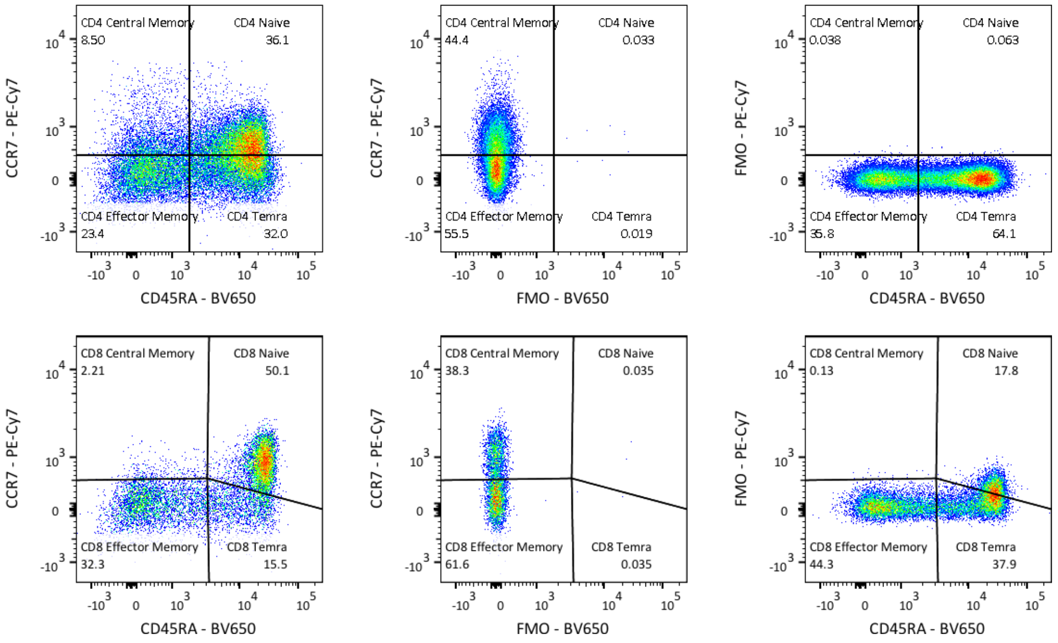

CD4 and CD8 Gating with CD62L Colour Map

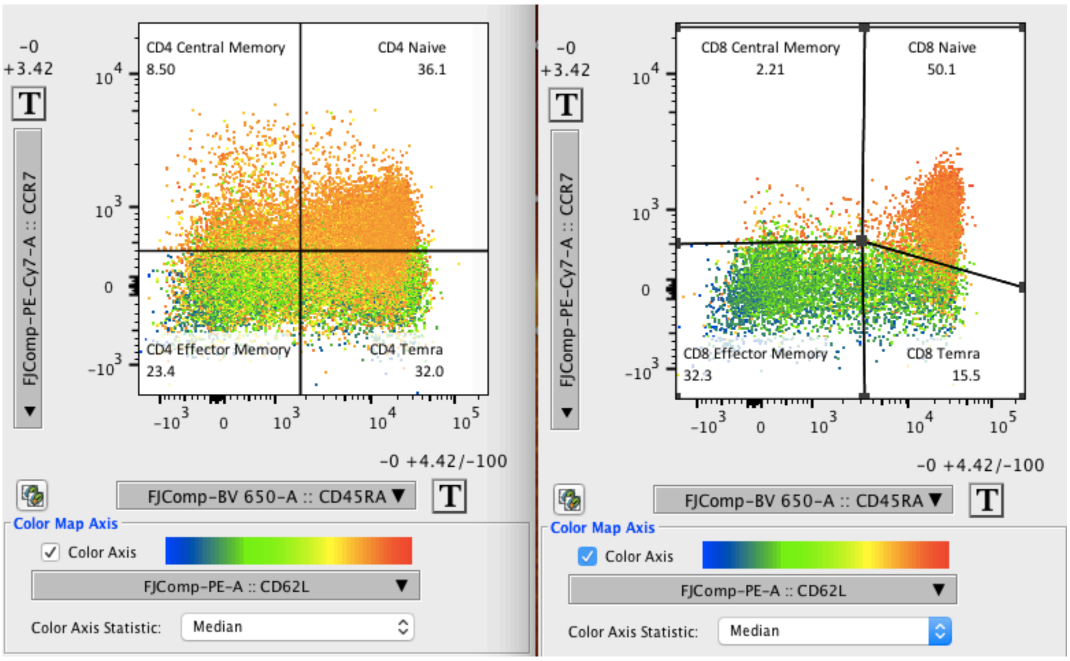

Supplementary Figure 3 – Tscm panel memory subset gating validation A representative compensation matrix, CCR7 and CD45RA fluorescence minus one dot plots, and CCR7/CD45RA gating with CD62L colour mapped to support definition of T-cell memory subset gates are shown.

Supplementary Information 1 – R code used to perform Spearman's correlation and generate correlograms

```
# Load packages required
library(readxl)
library(corrplot)
library(psych)

# Load data
resPP <- as.data.frame(read_excel("File Location"))

# Define columns for analysis
resPP3 <- resPP[,2:44]

# Run Spearman's correlation to generate correlation matrix
PPcor3 <- corr.test(resPP3,use="pairwise.complete.obs", method = "spearman", adjust = "none")

# Define p-values and r-values for corrplot
pmaT3 <- PPcor3$p
M3 <- PPcor3$r

# Generate correlogram using corrplot package
corrplot(M3[1:8,], order="original", col=colorRampPalette(c("blue","white","red"))(200), p.mat = pmaT3, sig.level = 0.05, insig="blank",tl.cex = 0.7, tl.col="black", addgrid.col="white")

# Save p-values and r-values as .csv files
write.csv(PPcor3$p, file = "PPcorPvaluenotadjusted.csv")
write.csv(PPcor3$r, file = "PPcorRvaluenotadjusted.csv")


# Repeat of procedure for second study group
resPnP <- as.data.frame(read_excel("File Location"))
resPnP3 <- resPnP[,2:43]
PnPcor3 <- corr.test(resPnP3,use="pairwise.complete.obs", method = "spearman", adjust = "none")
pmaT3PnP <- PnPcor3$p
M3PnP <- PnPcor3$r
corrplot(M3PnP[1:8,1:42], order="original", col=colorRampPalette(c("blue","white","red"))(200), p.mat = pmaT3PnP, sig.level = 0.05, insig="blank",tl.cex = 0.7, tl.col="black", addgrid.col="white")
write.csv(PnPcor3$p, file = "PnPcorPvaluenotadjusted.csv")
write.csv(PnPcor3$r, file = "PnPcorRvaluenotadjusted.csv")
```

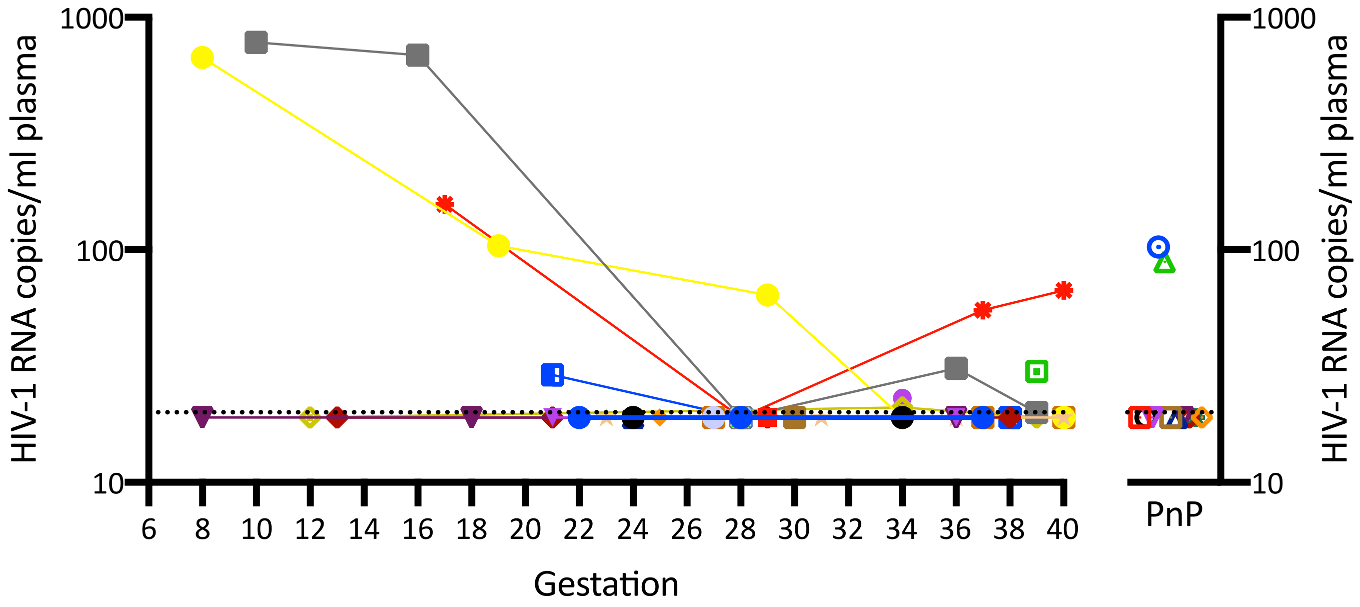

Supplementary Figure 4 – HIV-1 RNA plasma loads of both study groups This graph shows the HIV-1 RNA copies/ml plasma with gestation week for the pregnant group, and separated on the right hand side are the cross-sectional viral loads from the HIV-1 postive non-pregnant participants. Participant samples are individually shaped, coloured, and are connected by lines to show changes during their time on the study. The dotted line at 20 copies/ml plasma denotes the limit of detectability of the assay. Four pregnant women who had detectable HIV-1 plasma RNA became undetectable during longitudinal follow up, two of which became detectable again after 28 weeks gestation. Two of the non-pregnant group had detectable viral loads.

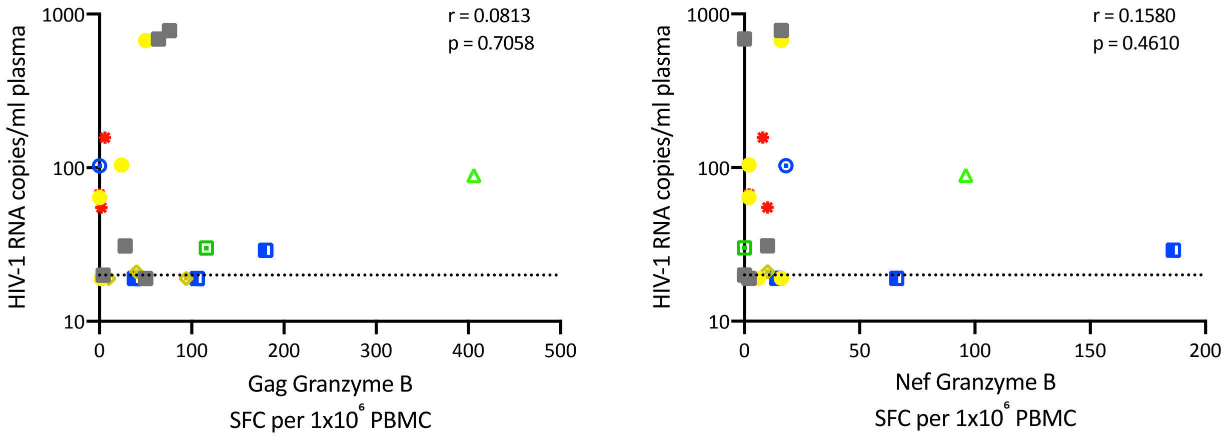

Supplementary Figure 5 – Gag and Nef granzyme B response correlations to plasma HIV-1 RNA of detectable study participants These graphs show Spearman's correlations of either Gag or Nef granzyme B responses against HIV-1 RNA. Study participants who had detectable plasma viral loads during the study from both pregnant and non-pregnant groups were combined to explore the potential association between their viral load and granzyme B responses. Participant samples are individually shaped and coloured. The dotted line at 20 copies/ml plasma denotes the limit of detectability of the assay. While Spearman's correlation cannot adjust for repeated measures from the same participants neither analysis identified an association between the viral load of study participants with detectable plasma HIV-1 RNA and either Gag or Nef granzyme B response.
